# Epigenetics and Autism

**DOI:** 10.1155/2013/826156

**Published:** 2013-09-15

**Authors:** Tafari Mbadiwe, Richard M. Millis

**Affiliations:** Department of Physiology & Biophysics, The Howard University College of Medicine, Washington, DC 20059, USA

## Abstract

This review identifies mechanisms for altering DNA-histone interactions of cell chromatin to upregulate or downregulate gene expression that could serve as epigenetic targets for therapeutic interventions in autism. DNA methyltransferases (DNMTs) can phosphorylate histone H3 at T6. Aided by protein kinase C**β**1, the DNMT lysine-specific demethylase-1 prevents demethylation of H3 at K4. During androgen-receptor-(AR-) dependent gene activation, this sequence may produce AR-dependent gene overactivation which may partly explain the male predominance of autism. AR-dependent gene overactivation in conjunction with a DNMT mechanism for methylating oxytocin receptors could produce high arousal inputs to the amygdala resulting in aberrant socialization, a prime characteristic of autism. Dysregulation of histone methyltransferases and histone deacetylases (HDACs) associated with low activity of methyl CpG binding protein-2 at cytosine-guanine sites in genes may reduce the capacity for condensing chromatin and silencing genes in frontal cortex, a site characterized by decreased cortical interconnectivity in autistic subjects. HDAC1 inhibition can overactivate mRNA transcription, a putative mechanism for the increased number of cerebral cortical columns and local frontal cortex hyperactivity in autistic individuals. These epigenetic mechanisms underlying male predominance, aberrant social interaction, and low functioning frontal cortex may be novel targets for autism prevention and treatment strategies.

## 1. Introduction

 Autism spectrum disorders (ASDs) are a range of neurodevelopmental disorders typically characterized by repetitive and stereotyped behavior, limited social development, and impaired language skills. Autistic disorder, Asperger syndrome, and pervasive development disorder not otherwise specified (PDD-NOS) are the most commonly diagnosed ASDs and there are also a large number of cases that are considered idiopathic because the etiology is unclear [1]. Notably, the incidence of ASD diagnoses has increased substantially in the past twenty years, growing by as much as 5- or 10-fold, although, the approximate 4 : 1 ratio of affected males to female has been maintained [2]. Some of this increase has been driven by shifting diagnostic criteria, heightened awareness, and improved diagnostic techniques [3]. However, some of this increase could be attributable to an authentic increase in the frequency of ASDs. The identity of the factors fueling this increase remains somewhat elusive and the precise causes of ASD diagnoses remain unknown [4]. Although its basis is thought to be multifactorial, autism is known to be a highly heritable disorder [5]. To a certain extent, the observed inheritance patterns can be explained by typical genetic processes but, especially in light of studies noting discordance among monozygotic twins, this is unlikely to be the whole story [6]. Recently, the idea that epigenetic influences may be partly responsible for the development of ASDs in many patients has gained popularity.

 Epigenetics refers to processes, notably the methylation of genes and modification of histones, that affect gene expression without altering the genetic code; a related term, epigenomics, concerns the study of the epigenome, which is a catalog of the heritable chemical changes made to DNA and histones. The effects of environment on phenotype are generally mediated through epigenetic processes [7]. Typical epigenetic mechanisms include the formation of 5-methylcytosine and acetylation of histones, thereby modifying the chromatin [8]. These epigenetic mechanisms can result in the silencing of particular genes and will ultimately impact the expressed phenotype [9]. Each individual's unique epigenome—the genome plus any epigenetic modifications—develops as a consequence of a variety of factors. The first, and probably most important, is the various effects exerted by the environment on the epigenome [10]. Second, the epigenome is itself heritable; a mother, for instance, can pass a methylated gene to her offspring [11]. Third, the epigenome—much like the genome—is subject to replication errors; however, whereas the typical error rate for gene replication is 1 : 1,000,000, the usual error rate for replicating epigenetic elements is closer to 1 : 1,000 [12]. Fourth, spontaneous changes to the epigenome, apart from changes driven by environmental causes, are thought to occur, known as epigenetic drift [13]. The latter two elements account for the stochastic nature of the epigenome that is the tendency of epigenomes to diverge despite having identical starting conditions. 

 This review examines the current state of knowledge concerning the impact of epigenetic mechanisms of the development of ASD and is organized in the following sections.

## 2. Epigenetic Protein-DNA Interactions: Proteins Mediating Epigenetic Signaling

### 2.1. MeCP2

Methyl CpG binding protein 2 (MeCP2) is active in CNS regulation and development of synaptic contacts [14]. MeCP2 is known to be involved in gene silencing and as a consequence in its role epigenetic regulation has been the focus of a significant amount of investigation [15]. The MeCP2 gene is located on the q arm of the X chromosome. Since the MeCP2 gene is located on the X chromosome, it is X-linked and subject to X inactivation. Until recently, it was widely thought that MeCP2 was only responsible for the silencing of genes [16]. However, gene silencing is inconsistent with the mode of action of the MeCP2 protein product. Like other members of the methyl-CpG binding domain (MBD) family, MeCP2 binds to methylated DNA [17] and, after binding, MeCP2 forms a complex with the enzyme histone deacetylase 1 (HDAC1) that removes acetyl groups from histones, thereby causing the chromatin structure to condense. The condensation of the chromatin is critical to gene inactivation. However, recent investigations suggest that MeCP2 may also be capable of acting as an activator of a variety of genes [18]. Although the mechanism of this activating action is not totally clear, the dual functionality of MeCP2 is amply demonstrated by studies showing that 63% of MeCP2-bound promoters are actively expressed [17]. It is not clear whether the role of MeCP2 in the epigenetic regulation of autism is related to its role as a gene silencer or promoter. Nevertheless, a correlation between reduced expression of MeCP2 and ASD is noted.

 Using immunofluorescence, Nagarajan et al. quantified MeCP2 in the frontal cortex (Brodmann area 9) and fusiform gyrus (Brodmann area 37) [19]. The frontal cortex had previously been linked to autism and associated with high levels of MeCP2 expression, whereas the fusiform gyrus is associated with face processing [20, 21]. MeCP2 expression at these brain sites was measured for 14 autistic brains, each of which was compared to three age-matched controls. In 11 of the 14 cases, the autism brain samples showed significantly decreased MeCP2 expression compared to age-matched controls; in some cases the reduction was as much as twofold. Similarly, the proportion of cells that expressed high levels of MeCP2 was reduced in 11 out of the 14 autistic samples. Of the six fusiform gyrus samples examined by Nagarajan et al., five showed decreased expression of MeCP2 in the fusiform cortex and each of those five were among the samples that exhibited decreased MeCP2 expression in the frontal cortex. This concordance suggests that whatever is responsible for decreased MeCP2 expression in the brains of ASD subjects is likely exerting a generalized, nonlocalized effect. The autism patients whose brains were examined by Nagarajan et al. were classified as idiopathic, indicating that there was no known genetic cause for their ASD or for the decreased MeCP2 expression that appears to be linked to the ASD. Epigenetic regulation might help explain these findings. In order to test for methylation of the promoter region associated with the MeCP2 gene, Nagarajan et al. conducted bisulfite sequencing. The MeCP2 is on the X chromosome, and all the study subjects were males and, thus, actively expressing the X chromosome. Methylation of the 5′ portion of the MeCP2 regulatory region was observed for most autism samples and the autism group showed a statistically significant increase in methylation when compared to similarly aged control group samples. As expected, an inverse correlation was found between promoter region methylation and MeCP2 expression. These findings suggest that aberrant methylation may have resulted in decreased expression of MeCP2, which was associated with autism. It is also worth noting that there is a well-established relationship between MeCP2 defects and Rett syndrome (Rett is definitively diagnosed by evidence of such a defect) since, from a clinical standpoint, Rett is classified as an ASD.

## 3. Epigenetic DNA-Protein Interactions

### 3.1. Protein Kinase C Beta

Recent evidence suggests that a correlation exists between downregulation of the protein kinase C beta gene (PRKCB1) in the temporal lobe and ASDs [22]. In particular, this association appears to be linked to the alternative splicing of PRKCB1 isozymes fsI and betaII. In addition, PRKCB1 haplotypes are (statistically) significantly associated with autism. Moreover, whole genome expression analysis showed less coordinated expression of PKCB1-driven genes [22]. Phosphorylation of histone H3 at threonine 6 (H3T6) by protein kinase c beta-1 protein appears to prevent lysine-specific demethylase 1 (LSD1) from demethylating H3K4 during androgen receptor-dependent gene activation [23]. This finding may partly explain the male predominance of ASDs and might support the hypothesis that the higher fetal androgen levels in males than females produce greater arousal inputs to the amygdala which might sensitize boys to environmental stressors. Girls lack such androgen-facilitated arousal inputs to the amygdala and are protected from such high arousal inputs by estrogens, oxytocin, and the oxytocin receptor [24]. A role for an oxytocin receptor polymorphism in ASDs is also reported in Chinese and Japanese cohorts [25, 26] and dysregulation of DNA methylation in the promoter region of the oxytocin receptor gene has been observed after acute psychosocial stress in an elderly German cohort [27].

### 3.2. Oxytocin Receptor

Epigenetic regulation of the oxytocin receptor gene (OXTR) has been implicated in the etiology of ASDs [28]. Oxytocin, along with vasopressin, has also been determined to have a prosocial function [29]. Insel was the first to suggest a link between oxytocin and ASDs [30]. Some evidence for this link comes from animal studies. *OXTR* and oxytocin-knockout mice have been shown to have limited social memory and a diminished ability to recognize other individuals, both of which are common ASD symptoms [31, 32]. Interestingly, the effect of OXTR-knockout on social functioning is thought to be sex-specific; both developmental compensation and the effects of vasopressin have been posited as possible explanations for the normal social development in female OXTR knockout mice [33]. These findings suggest that any defect of the oxytocin pathway, including a deficiency of oxytocin receptors, may in some cases contribute to the development of ASDs. A diminished number of oxytocin receptors can have a variety of causes, including both genomic and epigenetic. A study by Gregory et al. looked at a family in which the mother had a hemizygous deletion of the OXTR gene, which she passed down to one of her sons, but not the other; however, both sons were diagnosed with autism [34]. Kimura et al. hypothesized that the promoter region of the OXTR gene of the affected sibling without the deletion was hypermethylated. Prior studies had identified two CpG island regions of the OXTR gene that, as a consequence of variable methylation, are reported to be associated with differential OXTR expression in liver and myometrium [35]. The first CpG island overlaps with exons 1, 2, and 3 of OXTR gene and the second CpG island was localized to the third intron. The second CpG island, within intron 3, was found to be heavily methylated in all three family members studied, the mother and her two affected sons. On the other hand, the other CpG island—overlapping exons 1, 2, and 3—was methylated differently in each of the family members; specifically, the affected sibling without the deletion showed significantly more methylation than his brother or mother at three sites within the intron. This hypermethylation occurred at locations that have previously been shown to impact OXTR expression. Since both siblings were autistic, even though one had a genomic deletion and the other displayed hypermethylated promoter regions, the Gregory et al. study stands as an elegant demonstration of the idea that epigenetic and genetic mechanisms can have equivalent effects on phenotype. The Gregory et al. study went one step further in an attempt to demonstrate that OXTR gene silencing is not unique to the highlighted case and is, in fact, a common contributor to autism. Five differentially methylated CpG islands were examined in a group of 20 autistic and 20 phenotypically normal individuals and, as expected, the autism group showed a statistically significantly higher level of methylation at several examined loci. These observations were made in samples of both blood and cerebral cortex. Additionally, low levels of OXTR expression were found to be associated with increased methylation at a statistically significant level. This finding strengthens the idea that promoter region methylation causes gene silencing. Moreover, when the data were stratified by sex, two of the loci showed significant differences in methylation for males only, thereby implying that the different frequencies of autism in males and females might be driven by epigenetic mechanisms. 

### 3.3. Bcl-2

 Bcl-2 (B-cell lymphoma 2) is a protein responsible for the regulation of apoptosis [36]. The *Bcl-2* gene has been implicated in the etiology of several cancers and the abnormal expression of the gene has also been linked to diseases with social impacts such as schizophrenia and autism [37]. The Bcl-2 protein is reported to be decreased in both the cerebellum and frontal cortex of autistic subjects compared to age and gender-matched controls [38, 39]. 

 Evidence linking *Bcl-2* gene expression to the development of ASD is still being assembled and thus the extent to which a causative relationship exists remains largely a matter of speculation. That said, a study by Nguyen et al. [40] examining lymphoblastoid cell lines from sets of monozygotic twins that were discordant for autism, and also comparing the twins' cell lines to those of their nonautistic, nontwin siblings, provides a basis for preliminary discussion.

### 3.4. RORA

 The proposition that epigenetic regulation of the retinoic acid-related orphan receptor alpha (RORA) might cause autism is relatively new. Although the functions of RORA are largely unknown, RORA regulation of circadian rhythm and neuroprotection against oxidative stress and inflammation is reported [41, 42]. A link between RORA and autism makes intuitive sense because autism is thought to be associated with increased levels of oxidative stress and inflammation [43, 44]. The Nguyen et al. study was the first to give scientific grounding to this intuition by noting that—as was the case with Bcl-2—there were statistically significant differences in both RORA gene promoter region methylation and protein product expression between autistic subjects and their (non-twin) unaffected siblings [40]. This was the case in both lympoblastoid cell line and postmortem brain tissue. Interestingly, when the population was stratified by the various ASD subtypes, it turned out that reduced RORA expression was only observed in ASD subjects with severe language impairment. As a consequence, reduced RORA expression was not observed in all ASD subjects. The notion that RORA methylation might be largely responsible for the language deficits sometimes associated with ASD is a useful finding that helps clarify the etiology of autism and an important first step in determining the particular mechanism responsible for these symphtoms. Moreover, the connection between promoter region methylation and RORA expression was confirmed by treatment with global inhibition of methylation using 5-Aza, which increased gene expression in autistic subjects, but not in unaffected subjects. However, as with Bcl-2, the effects of 5-Aza on undiagnosed cotwins and unaffected subjects were not found to be statistically significant. This finding must be interpreted cautiously because there could be a variety of possible explanations.

### 3.5. *β*-Catenin

 The *β*-catenin gene has been the subject of much investigation related to its potential as an oncogene but it can also act as the fulcrum in at least two relevant epigenetic processes related to the development of ASD. One epigenetic process of *β*-catenin involves estrogens. Estrogens are critical players in the sexual differentiation of the brain and it is likely that brain estrogen levels are increased in autistic subjects [45]. Estrogens, being steroid hormones, and their receptors—including estrogen receptor alpha (ER*α*)—are located in the nucleus and in the cytosol of target cells. One of the targets of cytosolic ER*α* is GSK3B, which is known to form a complex with *β*-catenin for degradation of *β*-catenin. ER*α* activation by estradiol is reported to release *β*-catenin from this complex, thereby increasing *β*-catenin availability [46]. An increase in the cytosolic concentration of estrogens is thought to result in increases in cytosolic and nuclear *β*-catenin during critical periods of prenatal and/or neonatal development wherein *β*-catenin binding to the LEC/TCF promoter has positive effects on Wnt pathway gene transcription. Such increased transcription in the Wnt pathway is strongly associated with the development of ASDs. The effect of ER*α* in this process is to cause the dissociation of *β*-catenin from a complex whose integral members include the proteins GSK3*β*, axin, and adenomatous polyposis coli tumor suppressor (APC). GSK3*β*, axin, and APC are negative regulators of the Wnt signaling pathway and the complex requires all of these constituents to initiate the destruction of *β*-catenin. The absence or downregulation of any of these components may increase the availability of cytosolic *β*-catenin, as well as in the various knockin effects discussed previously—increased nuclear *β*-catenin with greater Wnt pathway transcription. Lithium, used mostly as a mood stabilizing drug, exerts an inhibitory effect on GSK3*β* both directly and indirectly, by interrupting the dephosphorylation of phospo-GSK3*β* [47]. In either case, the effect is the same, and also the same as that of increased estrogen levels; that is, the complex responsible for initiating the degradation of *β*-catenin is made nonfunctional, and the concentration of cytosolic *β*-catenin increases.

### 3.6. The Neurexin-Neuroligin Pathway

SHANK3 is a scaffolding protein in the neurexin-neuroligin pathway that interacts with synaptic proteins. Recent research suggests that copy number variations or mutations of either of these proteins may be associated with the development of ASDs [48]. It appears that epigenetic mechanisms are used to control the expression of this gene. For instance, Beri et al. identified five CpG islands in the SHANK3 gene, the posttranslational methylation of which determines gene expression [49]. One specific locus—CpG island 2—appeared to particularly impact tissue SHANK expression. Further more, taking advantage of the fact that the SHANK3 gene is well conserved between humans and rodents, Uchino and Waga demonstrated that the neonatal expression of certain SHANK3 transcripts in mice temporarily decreases as the methylation of CpG island 2 peaks two weeks after birth [50]. This suggests that the expression of SHANK3 (and thus its effect on the development of ASD) is regulated by epigenetic mechanisms, though this connection has yet to be directly established in humans. Additionally, two genes responsible for the production of cell adhesion molecules in this pathway, NLGN3 and NLGN4, have also been associated with the development of ASD [48]. However, to date, epigenetic regulation of this gene is still unproven [51].

## 4. Role of Maternal Hypomethylation in Autism

Thus far, discussion of the contribution made by epigenetic mechanisms to the development of ASDs has focused on the genes and proteins of autistic patients. There is good reason for this. ASDs are known to possess a variety of genetic determinants, so investigations into the effects of gene silencing or promotion naturally focus on the silencing or promotion of the genes of the patient. However, epigenetic mechanisms do not exert their influence only by mechanisms of gene manipulation. For example, epigenetic influences on the maternal genome could alter the intrauterine environment such that the probability of the offspring developing ASDs is increased or decreased. DNA hypomethylation linked to variants in the maternal folate pathway has been linked to aberrant fetal development [52, 53]. Also, and more importantly, folate is the primary one carbon donor critical for methylation reactions. Because epigenetic mechanisms typically exert their influences by methylation of DNA, investigation of the folate pathway should provide insight into the availability of methylation precursors and also the extent of genomic methylation in mothers of both their autistic and their unaffected children. 

 A study by James et al. was performed, in part, to bolster the findings that mothers of autistic children often presented with aberrant DNA methylation [54]. Mothers of autistic children exhibited significantly lower levels of methylfolate and methionine—essential precursors for DNA methylation—than their counterparts in the control group. In addition, levels of the methylation-inhibiting proteins S-adenosylmethionine, adenosine, and homocysteine were all elevated in autism mothers. S-adenosylmethionine (SAM) is the primary methyl donor for the DNA methyltransferase reaction, which produces S-adenosylhomocysteine (SAH) and methylated DNA. Because SAM and SAH are linked by the transferase reaction, the SAM/SAH ratio is generally considered to be a good indicator of DNA methylation potential. Mothers of autistic children displayed a lower SAM/SAH ratio than the control group, which is indicative of a diminished capacity for methylation. Going one step further, in addition to having a lower capacity for methylation, the DNA of autism mothers is in fact less methylated than that of mothers of unaffected children; the ratio of 5-methylcytosine to total cytosine—a measure of overall genomic methylation—was significantly lower in the mothers of autistic children. Taken together, this evidence strongly suggests that hypomethylation of maternal DNA may be linked to ASDs. The significance of these findings about the influences of epigenetics on the development of autism is not entirely clear. However, it is not even clear that what is being observed is the effect of epigenetics. For example, although the ratio of 5-methycytosine to total cytosine was statistically correlated to the SAM/SAH ratio, it was more closely linked to the presence of an uncommon recessive allele in the gene that codes for reduced folate carrier protein. It is generally assumed that genes subjected to an atypical level of methylation are manifesting the effects of epigenetic influences. However, for this particular case of the SAM/SAH ratio, this assumption should be questioned. If, as the correlation data suggest, the differential methylation exhibited by mothers of autistic children is largely a consequence of a genetic polymorphism, then it is not likely to be epigenetics at work. If intrauterine conditions are altered by maternal hypomethylation, such changed conditions must impact fetal development pathways by specific mechanisms that remain to be elucidated.

## 5. Epigenetics and Nutritional Factors in Autism

 The period of development in which the nutritional imbalance occurs is very important in determining which disease-related genes will be affected. Different organs have critical developmental stages, and the time point at which they are compromised will predispose individuals to specific diseases. Epigenetic modifications that occur during development may not be expressed until later in life depending on the function of the gene. While the majority of studies implicate prenatal periods as critical time windows, some research has shown that nutritional intake during adulthood can also affect the epigenome.

 Genetic polymorphisms of cytochrome P450 enzymes have been linked to ASD, specifically the cytochrome P450 family 27 subfamily B gene (CYP27B1) that is essential for proper vitamin D metabolism. Epigenetic regulation of cytochrome P450 genes for hydroxylation and activation of vitamin D in has been shown in prostate cancer cells [55]. Vitamin D is important for neuronal growth and neurodevelopment, and defects in metabolism or deficiency have also been implicated in ASDs [56]. Mutations of MeCP2 associated with impaired methylation are known to be associated with ASDs and the related neurological disorder, Rett syndrome. One component of Rett syndrome is abnormal bone formation wherein abnormal vitamin D metabolism is associated with epigenetic dysregulation of cytochrome P450 genes [57] which could be a conceptual model for epigenetic interactions between MeCP2, vitamin D, and cytochrome P450 genes [56]. Abnormal folic acid metabolism may also play a role in the decreased capacity for methylation and DNA hypomethylation associated with significantly greater than normal levels of plasma homocysteine, adenosine, and SAH in mothers of subjects diagnosed with ASDs [58]. Changes in autism-related behaviors are reported to be strongly associated with vitamin-supplementation associated changes in plasma levels of biotin and vitamin K [59] and although biotin is a known cofactor in bioavailability of methyl groups for DNA methylation, a vitamin-K-related epigenetic mechanism has not been described. 

## 6. Epigenetics and Toxic Factors in Autism

### 6.1. Valproic Acid

 Valproic acid (VPA) is a therapeutic anticonvulsant and mood stabilizing drug that gained attention in the 1980s as a potential teratogen. VPA exposure is highly correlated with autism; as many as 60% of infants who exhibit the suite of symptoms associated with VPA teratogenicity also display two or more autistic characteristics [60]. Autism has also been shown to occur in 9% of cases of prenatal exposure to VPA [61]. The mechanisms underlying the pharmacological actions of VPA are also suggestive of a correlation between VPA and autism [62]. VPA is responsible for inhibiting two enzymes: myo-inositol-1-phosphate (MIP) synthase and the class 1 and 2 histone deacetylase (HDAC). HDAC1 is an important inhibitor of DNA transcription that works by associating with the LEC/TCF transcription factor. When HDAC1 is removed from the LEC/TCF complex, it leaves behind a primed (but inactive) promoter of gene transcription. The primed promoter then forms a complex with *β*-catenin, thus activating the promoter and increasing transcriptions rates of a variety of genes in the Wnt signaling pathway including cyclin D1, required for the transition from the G1 to S phases of mitosis, and MYC, a transcription enhancer for many genes throughout the genome [63]. Accordingly, the consequence of VPA-mediated inhibition of HDAC1 is to upregulate the transcription of Wnt pathway genes. In addition, VPA increases cellular levels of *β*-catenin, presumably in response to the increased availability of primed LEC/TCF promoters [64]. The effect of VPA on Wnt gene transcription is well understood but fails to explain the connection between VPA and autism. In order to complete this link, an increase in the number of neocortical minicolumns is highly correlated with autism [65]. This observation is supported by fMRI studies that report differences in how autism brains coordinate the processing of information [66]. It is reasonable to assume that processes which upregulate genes of the Wnt signaling pathway—such as prenatal exposure to VPA—may result in poorly regulated mitosis and cellular proliferation, one manifestation of which could be an increase in the number of neocortical minicolumns and macrocephaly. This mechanism has been observed at work in a slightly different context. Recall that MeCP2 inactivates genes by forming complexes with a variety of different molecules. One of these molecules is HDAC1 [67] and in the absence of HDAC1, or even if HDAC1 has merely been downregulated, the gene inactivating properties of MeCP2 is expected to have a diminished effect. One of the promoters on which MeCP2 typically exerts its regulatory effect is the LEC/TCF promoter, which, as mentioned previously, ultimately regulates the transcription rates of the Wnt signaling pathway. Although MeCP2 has effects on gene methylation, the function of HDAC1 concerns acetylation of histones. However, to transcriptionally deactivate genes, they must often be both methylated and deacetylated. Thus, VPA-induced inhibition of HDAC1 interferes with the functionality of MeCP2 which appears to increase the risk of developing ASDs. 

## 7. Epigenetics and Other Illnesses Associated with Autism

Epigenetic effects may also manifest through aberrant methylation patterns of imprinted genes. The expression of imprinted genes, which are mostly found in clusters on chromosomes 6, 7, 11, 14, and 15, is controlled by a series of epigenetic marks (DNA methylation and histone modification). Imprinting defects may be primary or secondary. Primary imprinting defects cause changes in methylation patterns but leave the DNA sequences unaltered and thus may be classified as an epigenetic mechanism [68]. Angelman syndrome, which is caused by an absence of active maternal genes in the 15q11-1q13 region, may result from a primary imprinting defect (though the syndrome is more commonly caused by a deletion on the maternal chromosome or a paternal uniparental disomy). There is some basis to suspect a link between Angelman syndrome and ASDs. For instance, a study by Steffenburg et al. sought to ascertain the frequency of ASDs among children diagnosed with Angelman syndrome [69]. The study screened a series of mentally retarded children for Angelman syndrome and subsequently evaluated the children with Angelman syndrome for evidence of autism. Four out of the approximately 49,000 screened children were diagnosed with Angelman syndrome, and each of those four were found to demonstrate autistic behaviors. However, other studies place the rate of cooccurrence of ASDs and Angelman syndrome at a rate of as low as 2%. Taken together, it seems reasonable to assert that, to the extent that Angelman syndrome and ASDs are linked, the condition of some percentage of these patients will be related to an epigenetic primary imprinting defect. That said, the available evidence does not establish whether the epigenetic defect causing Angelman syndrome leads directly to autistic symptoms or if instead the relationship between Angelman syndrome and ASDs is merely correlative, and not causative.

 On the other hand, secondary imprinting defects occur when a gene mutation results in improper epigenetic regulation. Such a defect may occur in Prader-Willi syndrome, which is characterized by the lack of a paternal contribution at the 15q11-q13 locus. The specific mutation most commonly responsible for secondary imprinting Prader-Willi syndrome is a cis-acting defect of the imprinting regulatory center of the Prader-Willi gene [70]. Prader-Willi patients present with autistic behavior more frequently than Angelman syndrome patients; studies suggest that the frequency of ASDs co-occurrence with Prader-Willi syndrome is between 18% and 38% [71], though the causative nature of this relationship has not been established.

 Fragile X syndrome is the leading single-gene cause of autism accounting for as many at 5% of all cases [72]. As with Prader-Willi and Angelman syndromes, epigenetic mechanism can contribute to the development of the fragile X, which is characterized by the presence of 200 or more CGG repeats in the 5′ untranslated region of the FMR1 gene [73]. The resulting increased concentration of cytosine and guanine nucleotides causes the global methylation of not only the CGG-repeat region but also adjacent regions, which happen to include FMR1 promoter elements. 

## 8. Conclusions

**Figure 1:**
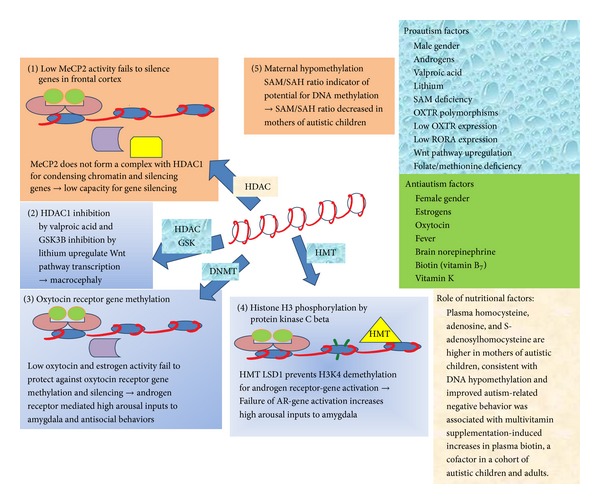
Main mechanisms of epigenetic alterations in autism. Each alteration involves many enzymes but the main players to cause methylation or acetylation are shown by arrows. These are not separate mechanisms and the enzymes do not act alone. Several enzymes act at a promoter simultaneously. (1) Low methyl CpG binding protein-2 (MeCP2) at CpG islands of frontal cortex reduces capacity for complexing with histone deacetylase 1 (HDAC1) for gene silencing. (2) HDAC1 inhibition by valproic acid exposure and glycogen synthetase kinase-3B (GSK3B) inhibition by lithium upregulate Wnt signaling pathway and activate transcription, associated with macrocephaly with increased number of cerebral cortical column. (3) DNA methyltransferase (DNMT) methylates oxytocin receptor gene produces low oxytocin and estrogen activity necessary for androgen receptor mediated high-arousal inputs to amygdala. (4) Histone H3 phosphorylation by protein kinase C beta activates the histone methyltransferase (HMT) lysine demethylase 1 (LSD1) which prevents demethylation of lysine-4 site of histone-3 (H3K4) that is also necessary for androgen receptor (AR) mediation of high arousal inputs to amygdala. (5) Maternal hypomethylation by dietary folic acid deficiency decreases availability of S-adenosyl methionine (SAM), associated with abnormal intrauterine growth.

Figure 1 summarizes the main epigenetic mechanisms that may play roles in ASD. Low activity of methyl CpG binding protein 2 (MeCP2) at CpG islands in genes of frontal cortex is shown to reduce the capacity for inhibiting HDAC1 and chromatin condensation for gene silencing. HDAC1 inhibition by valproic acid and GSK3*β* inhibition by lithium are shown to upregulate the Wnt signaling pathway which causes accumulation of *β*-catenin in the cytoplasm and its translocation to the nucleus, acting as an activator of transcription and resulting in macrocephaly with increased numbers of cerebral cortical columns. DNMT is shown to methylate the oxytocin receptor gene and silence it, resulting in the low oxytocin and estrogen activity necessary for androgen receptor mediation of high arousal inputs to the amygdala, associated with antisocial behaviors after exposure to environmental stressors. Histone H3 phosphorylation by protein kinase C beta is shown to activate the LSD1, an HMT that prevents demethylation of H3K4, that is also necessary for androgen receptor mediation of high arousal inputs to the amygdala. Hypomethylation by decreased availability of S-adenosyl methionine (SAM) is shown to occur in mothers of autistic children. Environmental and nutritional conditions acting as pro- or antiautism factors by epigenetic mechanisms suggest strategies for decreasing the prevalence of ASD. This knowledge of putative epigenetic targets should motivate clinical practitioners and educators to develop novel treatment strategies based on the environment-gene interactions which could contribute to the core symptoms of ASD.

## References

[1] Gillis RF, Rouleau GA (2011). The ongoing dissection of the genetic architecture of autistic spectrum disorder. *Molecular Autism*.

[2] Kadesjö B, Gillberg C, Hagberg B (1999). Brief report: autism and asperger syndrome in seven-year-old children: a total population study. *Journal of Autism and Developmental Disorders*.

[3] Levy SE, Mandell DS, Schultz RT (2009). Autism. *The Lancet*.

[4] Stoltenberg C, Schjølberg S, Bresnahan M (2010). The autism birth cohort: a paradigm for gene-environment-timing research. *Molecular Psychiatry*.

[5] Hu VW, Frank BC, Heine S, Lee NH, Quackenbush J (2006). Gene expression profiling of lymphoblastoid cell lines from monozygotic twins discordant in severity of autism reveals differential regulation of neurologically relevant genes. *BMC Genomics*.

[6] Ptak C, Petronis A (2010). Epigenetic approaches to psychiatric disorders. *Dialogues in clinical neuroscience*.

[7] Bell JT, Spector TD (2011). A twin approach to unraveling epigenetics. *Trends in Genetics*.

[8] Vanyushin BF (2005). Enzymatic DNA methylation is an epigenetic control for genetic functions of the cell. *Biochemistry*.

[9] Feinberg AP (2010). Epigenomics reveals a functional genome anatomy and a new approach to common disease. *Nature Biotechnology*.

[10] Jaenisch R, Bird A (2003). Epigenetic regulation of gene expression: how the genome integrates intrinsic and environmental signals. *Nature Genetics*.

[11] Hjelmeland LM (2011). Dark matters in AMD genetics: epigenetics and stochasticity. *Investigative Ophthalmology and Visual Science*.

[12] Riggs AD, Xiong Z, Wang L, LeBon JM (1998). Methylation dynamics, epigenetic fidelity and X chromosome structure. *Novartis Foundation Symposium*.

[13] Petronis A, Gottesman II, Kan P (2003). Monozygotic twins exhibit numerous epigenetic differences: clues to twin discordance?. *Schizophrenia Bulletin*.

[14] Luikenhuis S, Giacometti E, Beard CF, Jaenisch R (2004). Expression of MeCP2 in postmitotic neurons rescues Rett syndrome in mice. *Proceedings of the National Academy of Sciences of the United States of America*.

[15] Chahrour M, Sung YJ, Shaw C (2008). MeCP2, a key contributor to neurological disease, activates and represses transcription. *Science*.

[16] Cohen S, Zhou Z, Greenberg ME (2008). Activating a repressor. *Science*.

[17] Yasui DH, Peddada S, Bieda MC (2007). Integrated epigenomic analyses of neuronal MeCP2 reveal a role for long-range interaction with active genes. *Proceedings of the National Academy of Sciences of the United States of America*.

[18] Mehler MF (2008). Epigenetic principles and mechanisms underlying nervous system functions in health and disease. *Progress in Neurobiology*.

[19] Nagarajan RP, Hogart AR, Gwye Y, Martin MR, LaSalle JM (2006). Reduced MeCP2 expression is frequent in autism frontal cortex and correlates with aberrant MECP2 promoter methylation. *Epigenetics*.

[20] LaSalle JM, Goldstine J, Balmer D, Greco CM (2001). Quantitative localization of heterogeneous methyl-CpG-binding protein 2 (MeCP2) expression phenotypes in normal and Rett syndrome brain by laser scanning cytometry. *Human Molecular Genetics*.

[21] Pelphrey K, Adolphs R, Morris JP (2004). Neuroanatomical substrates of social cognition dysfunction in autism. *Mental Retardation and Developmental Disabilities Research Reviews*.

[22] Lintas C, Sacco R, Garbett K (2009). Involvement of the PRKCB1 gene in autistic disorder: significant genetic association and reduced neocortical gene expression. *Molecular Psychiatry*.

[23] Metzger E, Imhof A, Patel D (2010). Phosphorylation of histone H3T6 by PKCbeta(I) controls demethylation at histone H3K4. *Nature*.

[24] Pfaff DW, Rapin I, Goldman S (2011). Male predominance in autism: neuroendocrine influences on arousal and social anxiety. *Autism Research*.

[25] Wu S, Jia M, Ruan Y (2005). Positive association of the oxytocin receptor gene (OXTR) with autism in the Chinese Han population. *Biological Psychiatry*.

[26] Liu X, Kawamura Y, Shimada T (2010). Association of the oxytocin receptor (OXTR) gene polymorphisms with autism spectrum disorder (ASD) in the Japanese population. *Journal of Human Genetics*.

[27] Unternaehrer E, Luers P, Mill J (2012). Dynamic changes in DNA methylation of stress-associated genes (OXTR, BDNF) after acute psychosocial stress. *Translational Psychiatry*.

[28] Jacob S, Brune CW, Carter CS, Leventhal BL, Lord C, Cook EH (2007). Association of the oxytocin receptor gene (OXTR) in Caucasian children and adolescents with autism. *Neuroscience Letters*.

[29] Gouin J-P, Carter CS, Pournajafi-Nazarloo H (2010). Marital behavior, oxytocin, vasopressin, and wound healing. *Psychoneuroendocrinology*.

[30] Insel TR (1992). Oxytocin—a neuropeptide for affiliation: evidence from behavioral, receptor autoradiographic, and comparative studies. *Psychoneuroendocrinology*.

[31] Ferguson JN, Young LJ, Hearn EF, Matzuk MM, Insel TR, Winslow JT (2000). Social amnesia in mice lacking the oxytocin gene. *Nature Genetics*.

[32] Takayanagi Y, Yoshida M, Bielsky IF (2005). Pervasive social deficits, but normal parturition, in oxytocin receptor-deficient mice. *Proceedings of the National Academy of Sciences of the United States of America*.

[33] Sun L, Huang L, Nguyen P (2008). DNA methyltransferase 1 and 3B activate BAG-1 expression via recruitment of CTCFL/BORIS and modulation of promoter histone methylation. *Cancer Research*.

[34] Gregory SG, Connelly JJ, Towers AJ (2009). Genomic and epigenetic evidence for oxytocin receptor deficiency in autism. *BMC Medicine*.

[35] Kimura T, Saji F, Nishimori K (2003). Molecular regulation of the oxytocin receptor in peripheral organs. *Journal of Molecular Endocrinology*.

[36] Tsujimoto Y, Finger LR, Yunis J (1984). Cloning of the chromosome breakpoint of neoplastic B cells with the t(14;18) chromosome translocation. *Science*.

[37] Glantz LA, Gilmore JH, Lieberman JA, Jarskog LF (2006). Apoptotic mechanisms and the synaptic pathology of schizophrenia. *Schizophrenia Research*.

[38] Fatemi SH, Stary JM, Halt AR, Realmuto GR (2001). Dysregulation of reelin and Bcl-2 proteins in autistic cerebellum. *Journal of Autism and Developmental Disorders*.

[39] Hossein Fatemi S, Halt AR (2001). Altered levels of Bcl2 and p53 proteins in parietal cortex reflect deranged apoptotic regulation in autism. *Synapse*.

[40] Nguyen A, Rauch TA, Pfeifer GP, Hu VW (2010). Global methylation profiling of lymphoblastoid cell lines reveals epigenetic contributions to autism spectrum disorders and a novel autism candidate gene, RORA, whose protein product is reduced in autistic brain. *FASEB Journal*.

[41] Akashi M, Takumi T (2005). The orphan nuclear receptor ROR*α* regulates circadian transcription of the mammalian core-clock Bmal1. *Nature Structural & Molecular Biology*.

[42] Boukhtouche F, Vodjdani G, Jarvis CI (2006). Human retinoic acid receptor-related orphan receptor *α*1 overexpression protects neurones against oxidative stress-induced apoptosis. *Journal of Neurochemistry*.

[43] Pardo CA, Vargas DL, Zimmerman AW (2005). Immunity, neuroglia and neuroinflammation in autism. *International Review of Psychiatry*.

[44] Chauhan A, Chauhan V (2006). Oxidative stress in autism. *Pathophysiology*.

[45] MacLusky NJ, Clark AS, Naftolin F, Goldman-Rakic PS (1987). Estrogen formation in the mammalian brain: possible role of aromatase in sexual differentiation of the hippocampus and neocortex. *Steroids*.

[46] Cardona-Gomez P, Perez M, Avila J, Garcia-Segura LM, Wandosell F (2004). Estradiol inhibits GSK3 and regulates interaction of estrogen receptors, GSK3, and beta-catenin in the hippocampus. *Molecular and Cellular Neuroscience*.

[47] Jope RS (2003). Lithium and GSK-3: one inhibitor, two inhibitory actions, multiple outcomes. *Trends in Pharmacological Sciences*.

[48] Liu Y, Du Y, Liu W Lack of association between NLGN3, NLGN4, SHANK2 and SHANK3 gene variants and autism spectrum disorder in a Chinese population. *PLoS ONE*.

[49] Beri S, Tonna N, Menozzi G, Bonaglia MC, Sala C, Giorda R (2007). DNA methylation regulates tissue-speci.c expression of Shank3. *Journal of Neurochemistry*.

[50] Uchino S, Waga C (2013). SHANK3 as an autism spectrum disorder-associated gene. *Brain Development*.

[51] Yasuda Y, Hashimoto R, Yamamori H (2011). Gene expression analysis in lymphoblasts derived from patients with autism spectrum disorder. *Molecular Autism*.

[52] Beaudin AE, Perry CA, Stabler SP, Allen RH, Stover PJ (2012). Maternal Mthfd1 disruption impairs fetal growth but does not cause neural tube defects in mice. *American Journal of Clinical Nutrition*.

[53] Scholl TO, Johnson WG (2000). Folic acid: influence on the outcome of pregnancy. *American Journal of Clinical Nutrition*.

[54] Jill James S, Melnyk S, Jernigan S, Hubanks A, Rose S, Gaylor DW (2008). Abnormal transmethylation/transsulfuration metabolism and DNA hypomethylation among parents of children with autism. *Journal of Autism and Developmental Disorders*.

[55] Luo W, Karpf AR, Deeb KK (2010). Epigenetic regulation of vitamin D 24-hydroxylase/CYP24A1 in human prostate cancer. *Cancer Research*.

[56] Currenti SA (2010). Understanding and determining the etiology of autism. *Cellular and Molecular Neurobiology*.

[57] O’Connor RD, Zayzafoon M, Farach-Carson MC, Schanen NC (2009). Mecp2 deficiency decreases bone formation and reduces bone volume in a rodent model of Rett syndrome. *Bone*.

[58] James SJ, Melnyk S, Jernigan S (2010). A functional polymorphism in the reduced folate carrier gene and DNA hypomethylation in mothers of children with autism. *American Journal of Medical Genetics, Part B*.

[59] Adams JB, Audhya T, McDonough-Means S (2011). Effect of a vitamin/mineral supplement on children and adults with autism. *BMC Pediatrics*.

[60] Moore SJ, Turnpenny P, Quinn A (2000). A clinical study of 57 children with fetal anticonvulsant syndromes. *Journal of Medical Genetics*.

[61] Rasalam AD, Hailey H, Williams JHG (2005). Characteristics of fetal anticonvulsant syndrome associated autistic disorder. *Developmental Medicine and Child Neurology*.

[62] Shimshoni JA, Dalton EC, Jenkins A (2007). The effects of central nervous system-active valproic acid constitutional isomers, cyclopropyl analogs, and amide derivatives on neuronal growth cone behavior. *Molecular Pharmacology*.

[63] Billin AN, Thirlwell H, Ayer DE (2000). *β*-Catenin-histone deacetylase interactions regulate the transition of LEF1 from a transcriptional repressor to an activator. *Molecular and Cellular Biology*.

[64] Wang Z, Xu L, Zhu X (2010). Demethylation of specific Wnt/*β*-catenin pathway genes and its upregulation in rat brain induced by prenatal valproate exposure. *Anatomical Record*.

[65] Williams EL, Casanova MF (2010). Autism or autisms? Finding the lowest common denominator. *Boletín de la Asociación Médica de Puerto Rico*.

[66] Minshew NJ, Williams DL (2007). The new neurobiology of autism: cortex, connectivity, and neuronal organization. *Archives of Neurology*.

[67] Nan X, Ng H-H, Johnson CA (1998). Transcriptional repression by the methyl-CpG-binding protein MeCP2 involves a histone deacetylase complex. *Nature*.

[68] Gos M (2013). Epigenetic mechanisms of gene expression regulation in neurological diseases. *Acta Neurobiologica Experimentalis*.

[69] Steffenburg S, Gillberg CL, Steffenburg U, Kyllerman M (1996). Autism in Angelman syndrome: a population-based study. *Pediatric Neurology*.

[70] Dykens E, Shah B (2003). Psychiatric disorders in Prader-Willi syndrome: epidemiology and management. *CNS Drugs*.

[71] Veltman MWM, Thompson RJ, Roberts SE, Thomas NS, Whittington J, Bolton PF (2004). Prader-Willi syndrome: a study comparing deletion and uniparental disomy cases with reference to autism spectrum disorders. *European Child and Adolescent Psychiatry*.

[72] McLennan Y, Polussa J, Tassone F, Hagerman R (2011). Fragile X syndrome. *Current Genomics*.

[73] Willemsen R, Levenga J, Oostra B (2011). CGG repeat in the FMR1 gene: size matters. *Clinical Genetics*.

